# Cell autonomous expression of inflammatory genes in biologically aged fibroblasts associated with elevated NF-kappaB activity

**DOI:** 10.1186/1742-4933-5-5

**Published:** 2008-07-16

**Authors:** Andres Kriete, Kelli L Mayo, Nirupama Yalamanchili, William Beggs, Patrick Bender, Csaba Kari, Ulrich Rodeck

**Affiliations:** 1 School of Biomedical Engineering, Science and Health Systems, Drexel University, Bossone Research Center, 3141 Chestnut Street, Philadelphia, PA 19104, USA; 2 Coriell Institute for Medical Research, 403 Haddon Avenue, Camden, NJ 08103, USA; 3 Dept. of Dermatology and Cutaneous Biology, Thomas Jefferson University, 233 South 10th Street, Suite 326 BLSB, Philadelphia, PA 19107, USA

## Abstract

**Background:**

Chronic inflammation is a well-known corollary of the aging process and is believed to significantly contribute to morbidity and mortality of many age-associated chronic diseases. However, the mechanisms that cause age-associated inflammatory changes are not well understood. Particularly, the contribution of cell stress responses to age-associated inflammation in 'non-inflammatory' cells remains poorly defined. The present cross-sectional study focused on differences in molecular signatures indicative of inflammatory states associated with biological aging of human fibroblasts from donors aged 22 to 92 years.

**Results:**

Gene expression profiling revealed elevated steady-state transcript levels consistent with a chronic inflammatory state in fibroblast cell-strains obtained from older donors. We also observed enhanced NF-κB DNA binding activity in a subset of strains, and the NF-κB profile correlated with mRNA expression levels characteristic of inflammatory processes, which include transcripts coding for cytokines, chemokines, components of the complement cascade and MHC molecules. This intrinsic low-grade inflammatory state, as it relates to aging, occurs in cultured cells irrespective of the presence of other cell types or the *in vivo *context.

**Conclusion:**

Our results are consistent with the view that constitutive activation of inflammatory pathways is a phenomenon prevalent in aged fibroblasts. It is possibly part of a cellular survival process in response to compromised mitochondrial function. Importantly, the inflammatory gene expression signature described here is cell autonomous, i.e. occurs in the absence of prototypical immune or pro-inflammatory cells, growth factors, or other inflammatory mediators.

## Background

Chronic inflammation associated with the aging process has been implicated in a host of degenerative disease states including osteoarthritis, atherosclerosis, type-2 diabetes and even cancer [1-3]. Age-associated chronic inflammatory states are distinct from inflammation triggered by infection. It is presently unclear to what extent chronic inflammatory states in older individuals represent autoimmune processes caused by deregulation of the immune system [4,5]. Alternatively, these states may arise as a consequence of an increased cell stress response in old cells triggered by molecular damage incurred over a lifetime. In support of cell autonomous causes for age-associated inflammation, expression of inflammatory markers, such as cytokines, has been observed in cells subjected to replicative senescence in vitro caused by serial passaging [6-9]. However, molecular events observed during replicative senescence *in vitro *do not necessarily mirror events that occur in human aging, which is of a dramatically different time frame. This consideration motivated the present investigation of age-associated changes in proliferating fibroblasts derived from donors at different biological ages. Only few reports using fibroblasts aged *in vivo *have been published and these reports largely focused on age-associated changes in cell cycle progression of dividing cells [10,11].

In contrast, in the present study we focused on 'inflammatory signatures', i.e. changes in gene expression patterns previously implicated in inflammatory states. Furthermore, we considered that determination of cell states associated with the aging process should be performed in quiescence rather than exponentially growing fibroblast cultures. This was based on the consideration that, under physiological conditions in tissues *in vivo*, the majority of fibroblasts neither proliferate nor have achieved replicative senescence akin to that of cultured fibroblasts. Therefore, we investigated differences in gene expression profiles of primary human fibroblasts derived from donors at different biological ages and rendered quiescent by growth factor starvation [12]. We report that expression of mRNA transcripts encoding proteins with roles in inflammation is elevated in fibroblasts derived from older individuals. This gene expression signature is associated with increased DNA binding of transcription factor nuclear factor kappa B (NF-κB) in a subset of aged cells and plays a role in mediating inflammatory responses.

## Methods

### Cell Lines and Culture Procedures

Human fibroblast cultures, established from skin samples derived from young and old donors, were obtained from the NIA Aging Cell Repository (Coriell Institute for Medical Research, Camden, NJ). All cell lines originated from 2 mm punch biopsies taken from the medial aspect of the upper arm. The donors were members of the Baltimore Longitudinal Study of Aging (BLSA) where they were characterized as "healthy" indviduals. The cell lines investigated had normal karyotypes. Coriell catalog numbers of these cell lines for the group of young donors were AG10803 (22 yrs), AG0454B (29 yrs), AG04441 (29-II yrs), AG13153 (30 yrs) and AG04438 (33 yrs), for the group of middle-age donors AG04456 (49 yrs), AG04659 (65 yrs), AG13369 (68-I yrs) and AG14251 (68-II yrs), and for the group of old donors AG11243 (74 yrs), AG09156 (81 yrs), AG13349 (86 yrs), AG13129 (89 yrs) and AG04064 (92 yrs). AG04456 (49 yrs) and AG14251 (68 yrs) were isogenic. Cells were grown in medium consisting of EMEM (Mediatech, Herndon, VA) supplemented with 2 mM L-glutamine and 15% FBS without antibiotics at 37°C and 5% CO_2 _according to Coriell's standard procedures. To avoid the influence of replicative senescence, cell lines selected for our cultures had undergone no more than half of the maximum population doublings at which previously determined senescence would occur. Twenty four hours prior to RNA collection, cells were placed in growth factor-free medium (MEM supplemented with 0.2% Bovine Serum Albumin/BSA). The protocol to prepare cells for microarray gene expression analysis was as follows: (Day 1) Cells were plated at 9000–12000 cells per cm^2 ^in regular growth medium; (Day 2) Cell culture medium was changed to growth-factor-free medium (EMEM with 0.2% BSA and 1% L-glutamine); (Day 3) Cells were washed with ice cold phosphate-buffered saline (PBS). Qiagen lysis buffer (RLT) was used to prepare cell lysates which were stored at -80°C. Cell cycle distribution was determined by PI staining followed by FACS analysis. Percentage of cells in S-phase before starvation was generally >10% and after mitogen starvation <1%.

### Microarray Analysis

RNA was isolated from the cell lysate using Qiagen RNeasy mini kit according to the manufacturer's instructions. Gene expression analysis was performed using the Codelink human bioarray containing single-stranded 30-mer oligonucleotide probes (Applied Microarrays, Tempe, AZ) and chips were run in duplicate. Details of this platform are available on the vendor's homepage website. Characteristics of the Codelink platform have been evaluated by us [13] and as part of the microarray quality control (MAQC) assessment [14]. Sample preparation and hybridization followed procedures described by Young *et al *[13]. Slides were scanned at 5 μm resolution with a ScanArray 4000 × l (Perkin Elmer, Waltham, Ma) and analyzed with the CodeLink Analysis Software, providing an integrated optical density (IOD) value for each hybridization spot, which is a measurement of an integrated background intensity value subtracted from the total pixel intensities within the area of the spot. Replicate readouts were averaged and normalized for differences between chips and outliers were detected. Expressions of characterized genes related to immunity and inflammation were identified and differential expressions determined. A variance filter trimmed the resulting list (p < 0.15). We used a correlative approach to search for similarities between the NF-κB profile and expression of genes related to inflammation. In modification of previously used rank correlation for template matching of phenotypical markers [15,16], we used Pearson correlation as a measure of similarity because the NF-κB values of the samples from young donors were close and within error margins, which can lead to low correlation values if ranked wrongly. Finally, the data was clustered by a dendrogram, using complete linkage analysis and a Canberra distance metric (J-Express, Molmine AS, Norway).

### NF-κB DNA Binding Activity Assay

We analyzed the DNA binding activity of NF-κB p65/RelA, a major component of the heterodimeric p50/RelA complex, with a chemiluminescent DNA binding assay in nuclear fractions. Cells were plated at subconfluency in regular growth medium. Twenty-four hours later, normal growth medium was replaced with a growth factor-free base medium for another 24 hours. On the day of sample collection, the cells (1.5 to 2 × 10^6^/sample) were washed once with PBS and trypsinized. They were centrifuged at 200–300 × g for 10 minutes and cell pellets were collected. The nuclear cell fractions were prepared using NE-PER^® ^Nuclear and Cytoplasmic Extraction Reagents (Pierce Biotechnology, Product No. 78833, Rockford, IL) according to the manufacturer's instructions. 10 μg of the nuclear cell fractions were used from each cell-line and their NF-κB p65 DNA binding activity was determined using the EZ-Detect NF-κB p65 Transcription Factor Kit (Pierce Biotechnology, Product No. 89859, Rockford, IL) according to the manufacturer's protocol. Two biological replicates of each sample were prepared and the signal of four readouts with a Veritas Microplate Luminometer was averaged.

## Results

**Figure 1:**
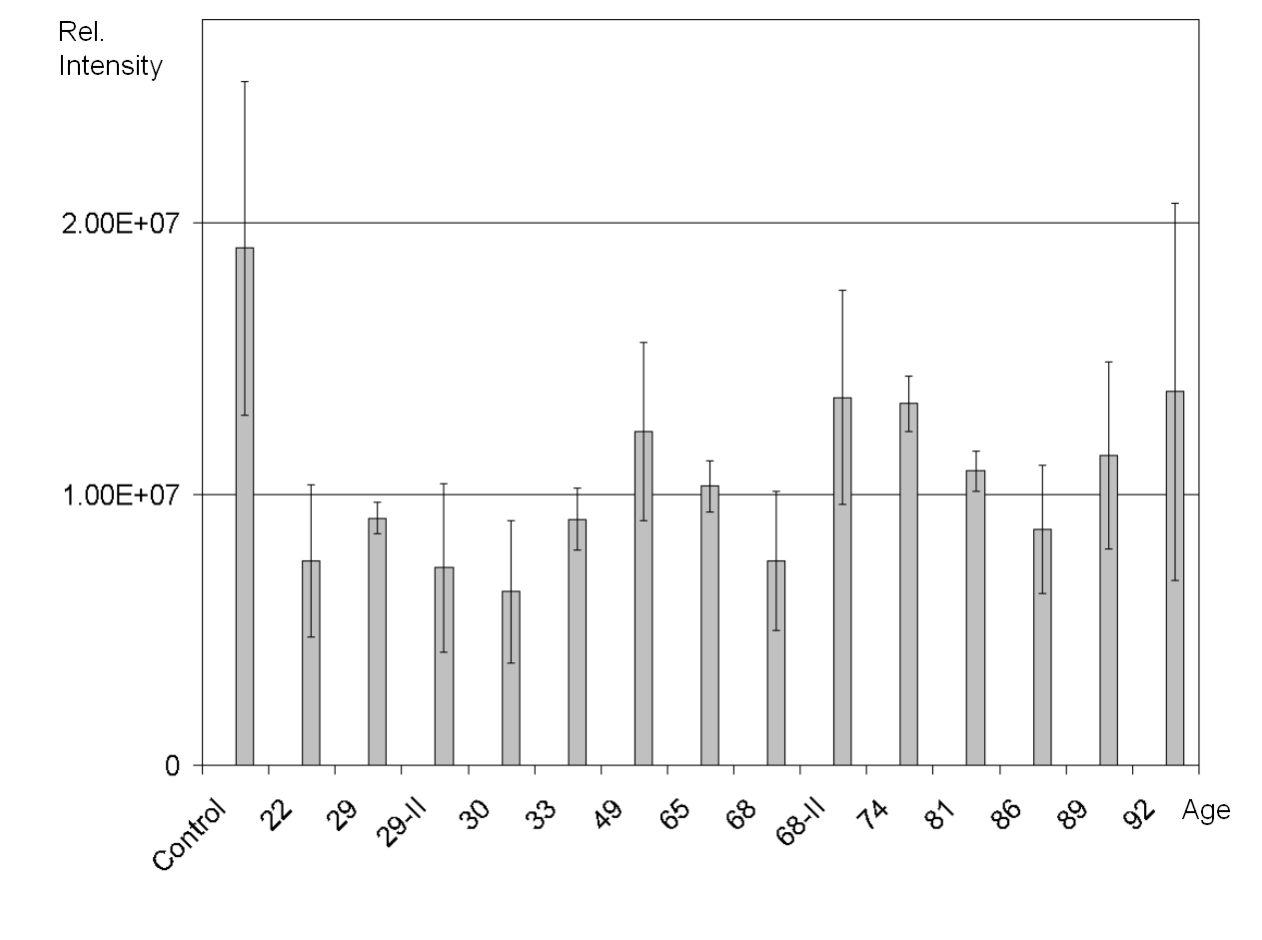
**NF-κB activity.** Shown are the results of a DNA binding assay of the NF-κB p65 transcription factor, using two biological replicates and four readouts for each sample. NF-κB activity, a key mediator of inflammation, is elevated in the nuclear fractions of fibroblasts from the group of older donors compared to young donors (p < 0.005). The DNA binding activity corresponds to a "low-grade" inflammatory state compared to the TNFα treated positive control.

**Figure 2:**
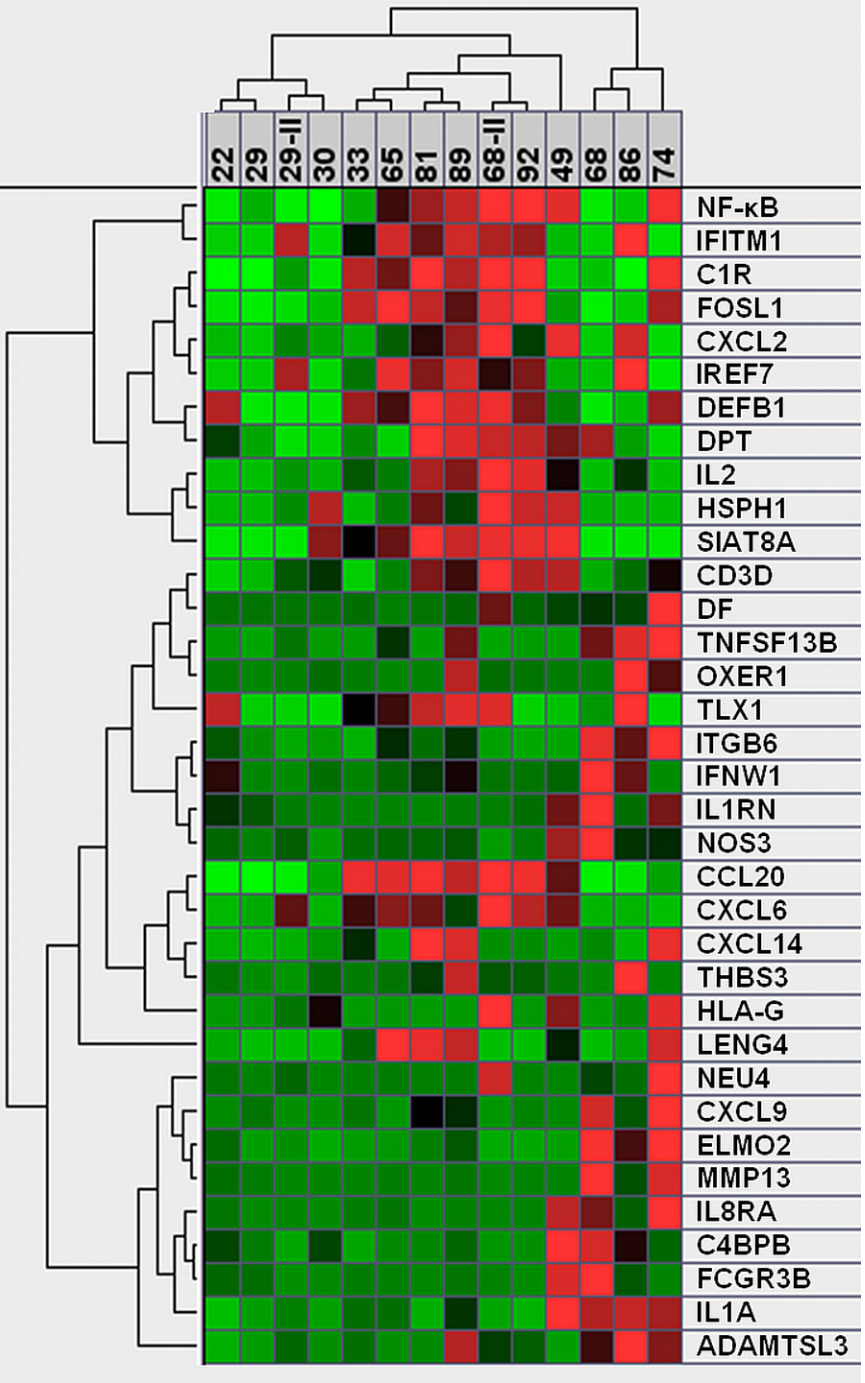
**Heatmap of gene-expression of inflammatory markers.** Green indicates repressed mRNA levels and red elevated levels. Intensities are normalized for each gene in each row. Samples and genes are grouped according to their similarity in profile, included is Nf-κB activity in the first row. The dendrogram reveals distinct clusters preferentially grouping cytokines, chemokines and interferon related molecules, specifically expressed in each cell strain. Samples of young donors are distinct from the group of older donors, and the samples of the 68 and 86 old donors are distinct within the group of older donors.

Previous efforts to identify age-associated changes in cellular homeostasis have largely relied on cells senesced *in vitro *or on the investigation of fast proliferating cells. In the present investigation, we focus on differences in quiescent fibroblasts derived from donors of different biological ages. Steady-state mRNA expression levels were determined by microarray analysis, as well as the NF-κB p65/RelA DNA binding assay in nuclear fractions. The NF-κB assay (Figure 1) revealed significantly higher binding activity in the middle-age (49–68 yrs) donors compared to the young (22–33 yrs) donors (p = 0.012, independent one-tailed test) as well as higher binding activity in the old age (72–92 yrs) donors compared to the young (p = 0.0039). However, the difference between the middle-age and old group was not significant (p = 0.34) and the combination of middle-age and old groups combined if compared to the young group gave a significant result (p = 0.004). The activity of the samples from the 68 and 86 year old donors were comparably low, and both of their gene expression profiles were different from those of the other older donors (Figure 2). The binding activity of NF-κB in middle-age and old donors was significantly higher than young donors and was considered to be moderate in comparison to the positive control (TNFα treated HeLa cells) (Figure 1).

In the microarray analysis, thirty-four genes, listed in table 1 and sorted by their similarity to the NF-κB activity, constituted an inflammatory signature in middle-age and older cells. Of these genes, 17 have a correlation factor R > 0.3 with the NF-κB profile. Genes representing a strong correlation with NF-κB included inflammatory genes such as FOS-like antigen 1 (*FOSL1*), antimicrobial peptide defensin beta one (*DEFB1*), T and B lymphocyte growth factor interleukin 2 (*IL2*), leukocyte modulator chemokine ligand 20 (*CCL20*) and cell adhesion molecule sialyltransferase 8A (*SIAT8A*). Elevated expression levels in cells from older individuals were observed for inflammatory cytokines including interleukin 1 receptor antagonist (*IL1RN*), interferon, omega 1 (*IFNW1*), interleukin 2 (*IL2*), interleukin 1 alpha (*IL1A*), interferon induced transmembrane protein 1 (9–27) (*IFITM1*), interferon regulatory factor 7 (*IRF7*), and interferon induced transmembrane protein 1 (*IFITM1*) (9–27). Expression of mRNA encoding proteins involved in the immune responses *in vivo *was elevated including leukocyte receptor cluster (*LRC*) member 4 (*LENG4*), D component of complement, adipsin (*DF*), tumor necrosis factor superfamily, member 13b (*TNFSF13B*), and Fc fragment of IgG, low affinity IIIb, receptor for CD16 (*FCGR3B*). A subset of cell strains contained higher message levels of inflammatory related genes such as matrix metalloproteinase-13 (*MMP-13*), integrin beta 6 gene (*ITGB6*), a cell surface receptor mediating cell-adhesion, a sialidase enzyme (*NEU-4*) and a MHC class I related gene (*HLA-G*) with roles in antigen presentation, and, potentially, autoimmunity [17-20]. A remarkable group of genes with roles in the inflammatory response were elements of both the classical and alternative complement cascades, including complement component 4 binding protein, beta (*C4BPB*), complement component 1, r subcomponent (*C1R*), and D component of complement, adipsin (*DF*), which recognize, initiate, and execute the destruction of antigenic molecules. Furthermore, elevated transcripts of several chemokine ligands were found (*CXCL2, CXCL9, CXCL14, CXCL20*) and chemokine ligand 6/granulocyte chemotactic protein 2 (*CXCL6*), consistent with the potential of aged fibroblasts to chemoattract leukocytes as well as other inflammatory-related molecules modulating the immune response.

**Table 1:** List of inflammatory genes. Given are accession numbers of genes, fold changes and p-values between the group of older and young donors, a Pearson correlation coefficient (R) expressing similarity to the NF-κB profile as seen in Figure 1, and a description of genes.

**Accession ID**	**Fold**	**p**	**R**	**Description**
NM_001733	2.3	0.023	0.74	complement component 1, r subcomponent (C1R)
NM_005438	2.0	0.033	0.71	FOS-like antigen 1 (FOSL1)
NM_000732	2.0	0.012	0.69	CD3D antigen, delta polypeptide (TiT3 complex) (CD3D)
NM_000586	4.6	0.020	0.68	interleukin 2 (IL2)
NM_004591	2.0	0.068	0.65	chemokine (C-C motif) ligand 20 (CCL20)
NM_005218	2.1	0.066	0.61	defensin, beta 1 (DEFB1)
NM_002993	2.0	0.097	0.58	chemokine (C-X-C motif) ligand 6 (granulocyte chemotactic protein 2) (CXCL6)
NM_003034	2.8	0.040	0.58	sialyltransferase 8A (GD3 synthase) (SIAT8A)
NM_002127	3.5	0.104	0.56	HLA-G histocompatibility antigen, class I, G (HLA-G)
NM_006644	2.6	0.102	0.52	heat shock 105 kDa/110 kDa protein 1 (HSPH1)
NM_080741	6.1	0.093	0.49	sialidase 4 (NEU4)
NM_002089	2.4	0.020	0.46	chemokine (C-X-C motif) ligand 2 (CXCL2)
NM_001928	5.6	0.094	0.45	D component of complement (adipsin) (DF)
NM_001937	2.2	0.017	0.41	dermatopontin (DPT)
NM_000634	4.3	0.068	0.39	interleukin 8 receptor, alpha (IL8RA)
NM_004887	3.5	0.047	0.38	chemokine (C-X-C motif) ligand 14 (CXCL14)
NM_024298	6.8	0.023	0.30	leukocyte receptor cluster (LRC) member 4 (LENG4)
NM_002416	3.6	0.068	0.22	chemokine (C-X-C motif) ligand 9 (CXCL9)
NM_006573	3.3	0.040	0.17	tumor necrosis factor (ligand) superfamily, member 13b (TNFSF13B)
NM_000575	2.7	0.033	0.17	interleukin 1, alpha (IL1A)
NM_003641	2.0	0.088	0.13	interferon induced transmembrane protein 1 (9–27) (IFITM1)
NM_000888	3.2	0.042	0.09	integrin, beta 6 (ITGB6)
NM_005521	2.0	0.094	0.06	T-cell leukemia, homeobox 1 (TLX1)
NM_004031	2.0	0.064	0.05	interferon regulatory factor 7 (IRF7), transcript variant d
NM_000716	2.2	0.085	0.05	complement component 4 binding protein, beta (C4BPB)
NM_007112	4.7	0.073	0.04	thrombospondin 3 (THBS3)
NM_182764	3.4	0.061	0.04	engulfment and cell motility 2 (ELMO2), transcript variant 3
NM_207517	2.7	0.041	0.01	ADAMTS-like 3 (ADAMTSL3)
NM_148962	4.8	0.083	-0.03	oxoeicosanoid (OXE) receptor 1 (OXER1)
NM_002427	5.1	0.093	-0.11	matrix metalloproteinase 13 (collagenase 3) (MMP13)
NM_000570	3.3	0.094	-0.15	Fc fragment of IgG, low affinity IIIb, receptor for (CD16) (FCGR3B)
NM_000577	2.7	0.116	-0.19	interleukin 1 receptor antagonist (IL1RN), transcript variant 3
NM_000603	2.4	0.088	-0.21	nitric oxide synthase 3 (endothelial cell) (NOS3)
NM_002177	2.3	0.127	-0.33	interferon, omega 1 (IFNW1)

## Discussion

Activation of the latent transcription factor NF-κB plays a key role in mediating inflammatory responses and gene expression patterns [21]. It has also been demonstrated that genetically blocking NF-κB in the skin of aged mice reverses the gene expression pattern seen in aged cells as well as reverts the appearance of tissues to that of younger skin samples, illustrating the critical role of NF-κB in the aging phenotype [22,23]. Here we show that in human aging NF-κB becomes constitutively active, mediating enhanced transcription of inflammatory markers. Cytokines, chemokines, and components of the complement cascade dominated the upregulated genes. This is the first report to demonstrate that a low-grade inflammatory state, as it relates to aging, occurs in cell lines irrespective of the presence of other cell types or the *in vivo *context, which highlights that this inflammatory state is intrinsic and cell autonomous. While NF-κB appears to be an important regulator, the profile of inflammatory genes expressing at higher levels is still specific for each sample investigated and not all genes are regulated by NF-κB, suggesting that additional factors may play a role mediating the profile.

It was reported that protein abundance of inflammatory markers parallels gene expression [24]; therefore it can be assumed that most inflammatory markers shown here would indeed contribute to the activation of the immune system, giving rise to a chronic inflammatory state *in vivo*. Specifically, fibroblasts acting as 'immune-competent' cells show higher levels of activity of molecules involved in antigen recognition, presentation and destruction such as *FGR3B, DF *and *C1R*. Essentially, aging cells send a destructive 'non-self' message to the immune system, for which rheumatoid arthritis is a prime example. The production of cytokines may establish a positive feedback loop since these markers can also activate NF-κB, and herewith increase and prolong chronic inflammation [25]. Similarly, interferons, known to be produced by synovial fibroblasts, have been associated with prolonged T cell survival in rheumatic joints [26].

Multiple factors can contribute to the activation and post-translational modification of NF-κB [21,27], but as it relates to aging, cell-intrinsic factors can be traced back to mitochondrial dysfunction. It has been recognized that mitochondrial deficiency accompanies the aging process, albeit the exact mechanisms for mitochondrial dysfunction discussed, for example mtDNA mutations in vertebrates, remain controversial [28-31]. Since we did not find indications for increased levels of reactive oxygen species (ROS) and related expression of scavenger molecules (data not shown), we hypothesize that activating mechanisms of NF-κB, as a consequence of mitochondrial dysfunction, may include other mechanisms such as accumulation of oxidized proteins and lipids in aged fibroblasts [32] or disturbances of intracellular calcium homeostasis [33,34]. Further, intracellular NADH abundance that has been related to defects in mitochondrial respiration is accompanied by inactivation of phosphatase and tensin homolg gene (*PTEN*). *PTEN *is a negative regulator of the Akt pathway [35,36], while Akt is known to converge with NF-κB signaling [35]. Insofar, NF-κB may participate in a retrograde response and cross-talk between dysfunctional mitochondria and nuclear genes as a cellular pro-survival mechanism. This would also explain why anti-inflammatory treatment regimes lead only to a temporary relief of symptoms, but would not correct for the primary cause of inflammation, which has its roots in cellular aging.

This study is a cross-sectional, rather a longitudinal study and the definition of age-groups follows the available samples in fulfillment of the stringent criteria set forth in the Method section. Variability as seen here is rooted in the process of biological aging, which is different from chronological age, and in cell selectivity during the initial establishment of cell cultures from biopsies. We also recognize that a study *in vitro *cannot capture the more complex interactions *in vivo*, involving other cellular participants and components of the immune system, which provoke, mediate, and amplify the primary cellular response. However, chronic inflammation has been implicated in many aging-associated conditions [1,37], and inflammatory markers described here have been reported in other age-related studies in which gene expression analysis was performed such as in brain [38], lung [39], liver [40], kidney [41] and coronary arteries [42]. Moreover, consensual findings show upregulation of NF-κB activity as a pivotal mediator of aging related inflammation in rodent and human tissues [22], including mouse brain, muscle [43-46] and human endothelial tissues [47], which suggests that similar processes as those observed here play a role in many other cell types and tissues.

## Conclusion

Overall this study provides evidence for a cell-intrinsic activation of NF-κB and related upregulation of inflammatory markers. While inflammation is a protective host response against harmful external stimuli, in the context of biological aging this once beneficial response becomes constitutively active, potentially as a consequence of mitochondrial dysfunction. This may in turn mediate susceptibility to age-related diseases in tissues since aberrant expression of inflammatory markers plays a key role in pathogenesis and tumorgenesis. Our study may provide a preliminary diagnostic tool to profile aging at the cellular level.

## Abbreviations

NADH: Nicotinamide adenosine dinucleotide hydrogen; *PTEN*: Phosphatase and tensin homolog gene; ROS: Reactive oxygen species; NF-κB: Transcription factor nuclear factor kappa B; TNF: Tumor necrosis factor.

## Competing interests

The authors declare that they have no competing interests.

## Authors' contributions

AK, KLM and UR designed research, NY, WB and CK performed research, AK, KLM, WB and PB analyzed data, AK, KLM and UR wrote the paper.
